# Arginine-Selective Bioconjugation Reagent for Effective ^18^F-labeling of Native Proteins

**DOI:** 10.1021/acs.jmedchem.4c00154

**Published:** 2024-03-13

**Authors:** Pragalath Sadasivam, Shivashankar Khanapur, Siddesh V. Hartimath, Boominathan Ramasamy, Peter Cheng, Chin Zan Feng, David Green, Christopher Davis, Julian L. Goggi, Edward G. Robins, Ran Yan

**Affiliations:** † School of Biomedical Engineering and Imaging Sciences, Department of Imaging Chemistry and Biology, King’s College, London SE1 7EH, U.K.; ‡ Institute of Bioengineering and Bioimaging, Agency for Science, Technology, and Research (A* STAR), 11 Biopolis Way, #01-02 Helios, Singapore 138667, Singapore; § Clinical Imaging Research Centre, 14 Medical Drive, #B01-01 Centre for Translational Medicine, Yong Loo Lin School of Medicine, National University of Singapore, Singapore 117599, Singapore; ∥ Molecular Imaging and Therapy Research Unit, South Australian Health, and Medical Research Institute (SAHMRI), North Terrace, Adelaide, SA 5000, Australia; ⊥ Adelaide Medical School, Faculty of Health and Medical Sciences, University of Adelaide, North Terrace & George Street, Adelaide, SA 5000, Australia; # Minerva Imaging ApS, Lyshøjvej 21, Ølstykke 3650, Denmark

## Abstract

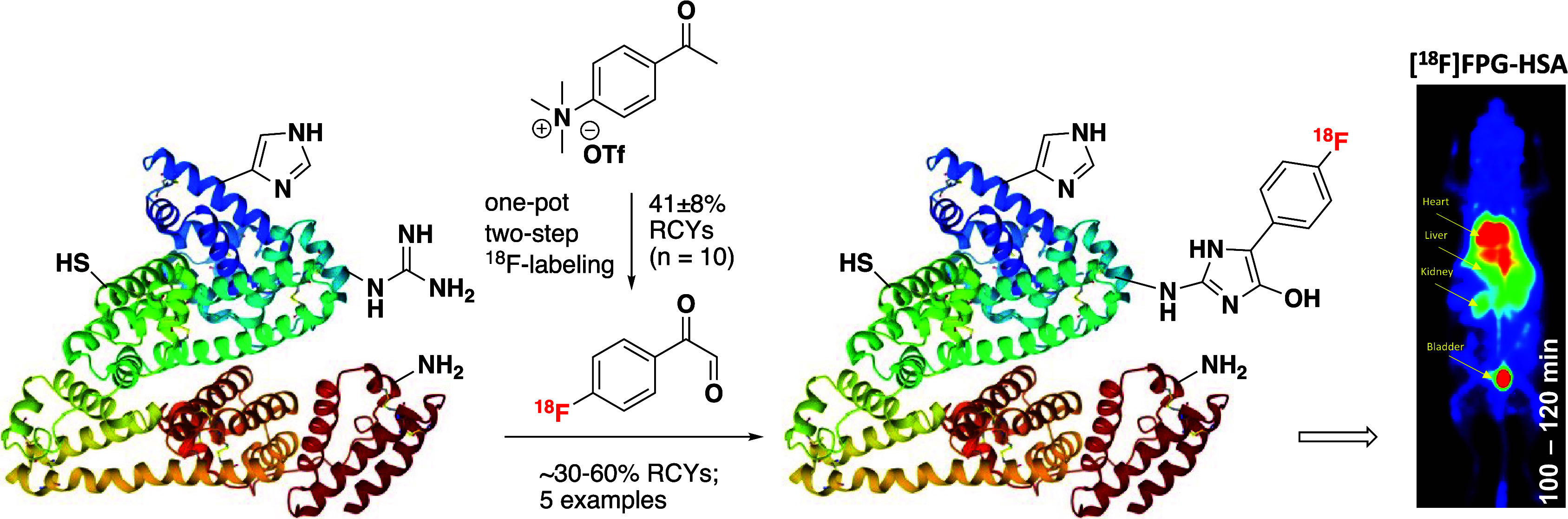

Protein-based ^18^F-PET tracers offer new possibilities in early disease detection and personalized medicine. Their development relies heavily on the availability and effectiveness of ^18^F-prosthetic groups. We prepared and evaluated a novel arginine-selective prosthetic group, 4-[^18^F]fluorophenylglyoxal ([^18^F]FPG). [^18^F]FPG was radiosynthesized by a one-pot, two-step procedure with a non-decay-corrected (n.d.c.) isolated radiochemical yield (RCY) of 41 ± 8% (*n* = 10). [^18^F]FPG constitutes a generic tool for ^18^F-labeling of various proteins, including human serum albumin (HSA), ubiquitin, interleukin-2, and interleukin-4 in ∼30–60% n.d.c. isolated RCYs. [^18^F]FPG conjugation with arginine residues is highly selective, even in the presence of a large excess of lysine, cysteine, and histidine. [^18^F]FPG protein conjugates are able to preserve the binding affinity of the native proteins while also demonstrating excellent *in vivo* stability. The [^18^F]FPG-HSA conjugate has prolonged blood retention, which can be applied as a potential blood pool PET imaging agent. Thus, [^18^F]FPG is an arginine-selective bioconjugation reagent that can be effectively used for the development of ^18^F-labeled protein radiopharmaceuticals.

## Introduction

Small functional proteins, such as diabodies, nanobodies, affibodies, and cytokines, etc., have emerged as prominent lead molecules for developing positron emission tomography (PET) tracers.^1,2^ Radiolabeled proteins typically display excellent binding affinity and specificity to their target receptors, which can enhance the precision and sensitivity of PET imaging and enable the detection and monitoring of pathological conditions with greater accuracy. Fluorine-18 is the most widely used radionuclide for PET imaging. It has ideal physicochemical properties, including a high positron yield of 97%, a short positron range of 0.5 mm in water, and a moderate half-life of *t*_1/2_ = 110 min, which is compatible with the clearance characteristics of many small proteins.^3^

A key challenge in developing protein-based ^18^F-PET tracers is to effectively radiolabel these delicate biomolecules with fluorine-18 under mild conditions. Efficient and selective conjugation methods are crucial to maintaining the pharmacological activities of the native proteins while ensuring optimal PET imaging performance.^4^ For this purpose, indirect labeling strategies using ^18^F-prothetic groups were developed to label native proteins.^4^ For example, active esters, such as *N*-succinimidyl-4-[^18^F]fluorobenzoate ([^18^F]SFB) and 2,3,5,6-tetrafluorophenyl 6-[^18^F]fluoronicotinate ([^18^F]TFPFN), modify the proteins at their lysine residues or the *N*-terminus.^5,6^ However, the standard radiosynthesis of [^18^F]SFB is a time-consuming three-step procedure.^5^ Despite several ^18^F-labeling strategies enabling one-step radiosynthesis of [^18^F]SFB,^7−9^ only two methods using either the spirocyclic iodonium ylide precursor or the highly toxic organotin precursor reported the decay-corrected isolated RCYs of [^18^F]SFB in 3–22 and 38–46%, respectively.^10,11^ On the other hand, [^18^F]TFPFN requires very high protein loading to achieve effective radiolabeling.^6^ Alternatively, the proteins are chemically modified or engineered with a functional group, such as hydrazine, (±)-H_3_RESCA, or cysteine. These modified proteins can be radiolabeled with 4-[^18^F]fluorobenzaldehyde (4-[^18^F]FBA), [^18^F]AlF, or *N*-[2-(4-[^18^F]fluorobenzamido)ethyl]maleimide (4-[^18^F]FBEM), respectively.^12−14^ However, for clinical use, the modified proteins need to be produced according to the stringent good manufacturing practice (GMP), which adds another significant barrier toward the clinical translation of the protein-based ^18^F-PET tracers. Thus, new ^18^F-prosthetic groups that can be rapidly radiosynthesized and effectively conjugated with native proteins are highly desirable for the preclinical development of new protein-based PET radiopharmaceuticals and can potentially aid in accelerating their clinical translation.

It has long been recognized that phenylglyoxals exhibit chemoselectivity and rapid kinetics toward the guanidium moiety of arginine residues in proteins.^15^ Recently, two fluorescent functionalized phenylglyoxals were reported for the arginine-selective bioconjugation of peptides and proteins. The resulting conjugates preserved the binding affinity of the native peptides and proteins.^16,17^ The promise of rapid and chemoselective bioconjugation toward the arginine residue provided the impetus for us to develop a fluorine-18 analogue of phenylglyoxal for bioconjugation of the low-molecular-weight proteins.

Herein, we report the radiochemical preparation of a novel ^18^F-prothetic group, [^18^F]fluorophenylglyoxal ([^18^F]FPG). We demonstrate that [^18^F]FPG can be effectively and chemoselectively coupled to various small proteins through arginine residues. The [^18^F]FPG protein conjugates exhibit excellent stability and preserve the binding affinity of the native proteins. We also demonstrate the potential PET imaging application of [^18^F]FPG-labeled human serum albumin (HSA) for blood pool imaging.

## Results

### Synthetic Chemistry and Radiochemistry

**Scheme 1:**
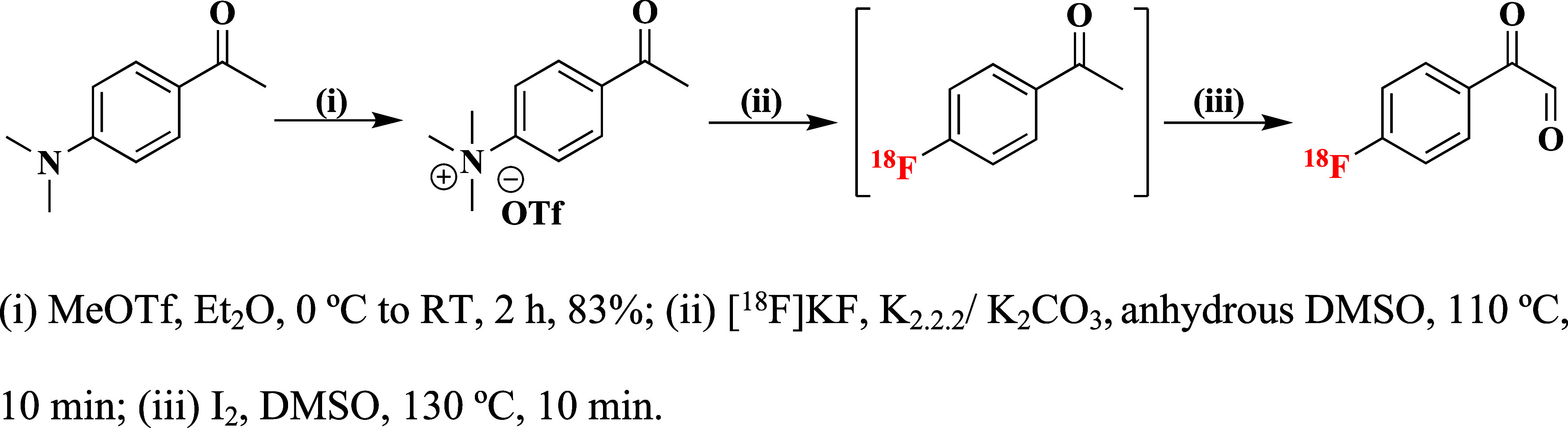
Preparation of the Radiolabeling Precursor and the One-Pot Two-Step Radiosynthesis of [^18^F]FPG

The radiolabeling precursor, 4-acetyl-*N*,*N*,*N*-trimethylbenzenammonium triflate, was prepared according to literature procedures by the methylation of 1-(4-(dimethylamino)phenyl)ethan-1-one with methyl trifluoromethanesulfonate in a good yield of 83% (Scheme 1).^18^

**Figure 1:**
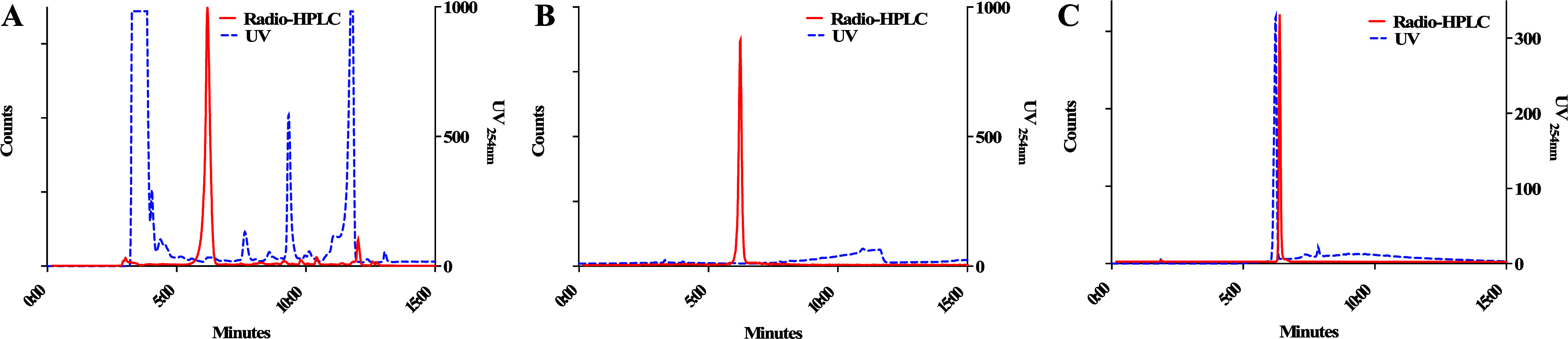
(A) HPLC chromatogram of the crude [^18^F]FPG radiolabeling reaction mixture; (B) HPLC chromatogram of the purified [^18^F]FPG; and (C) HPLC chromatogram of the purified [^18^F]FPG coinjected with its nonradioactive reference compound.

Subsequently, the 4-acetyl-*N*,*N*,*N*-trimethylbenzenammonium triflate was reacted with no-carrier-added (n.c.a.) fluoride-18 in the presence of K_2.2.2_/K_2_CO_3_ in anhydrous dimethyl sulfoxide (DMSO) at 110 °C for 10 min to produce the intermediate, [^18^F]fluoroacetophenone ([^18^F]FAcP) in a non-decay-corrected (n.c.d.) isolated RCY of 30 ± 8% (*n* = 6) starting from fluoride-18. The purified [^18^F]FAcP was used for the subsequent oxidation reaction to form [^18^F]FPG under different conditions as summarized in Table 1. First, the selenium-mediated oxidation^19^ of the carbonyl α-methylene position was attempted with H_2_SeO_3_ in 1,4-dioxane/H_2_O (20:1) (Table 1, entry 1–3). However, only around 60% of [^18^F]FAcP was converted to the desired product with H_2_SeO_3_ (100 mg, 776 μmol) (Table 1, entry 3). In contrast, when SeO_2_ (50 mg, 450 μmol) was used as an oxidant, [^18^F]FPG was produced in near-complete radiochemical conversion (RCC) at 110 °C in 20 min (Table 1, entry 6). However, preparation of [^18^F]FPG without purification of [^18^F]FAcP using SeO_2_ did not yield the desired [^18^F]FPG. To develop a one-pot procedure for [^18^F]FPG radiosynthesis, Kornblum oxidation^20,21^ was employed. Iodine (I_2_) was used to generate an α-iodoketone *in situ* followed by oxidation with DMSO to yield [^18^F]FPG. Excellent RCCs of 92 ± 4% (*n* = 3) were observed when 100 μmol of I_2_ was added directly to the crude reaction mixture of [^18^F]FAcP and heated at 130 °C for 10 min (Table 1, entry 7). This one-pot, two-step radiosynthesis produced [^18^F]FPG, after high performance liquid chromatography (HPLC) purification, in an n.d.c. isolated RCY of 41 ± 8% (*n* = 10) within 1 h starting from fluoride-18 (Figure 1A). The radiochemical purity of [^18^F]FPG was >99% (Figure 1B) with molar activities of 303 ± 104 GBq/μmol (*n* = 10) when starting from 7 to 10 GBq of fluoride-18. The [^18^F]FPG was coeluted with its nonradioactive reference compound (Figure 1C). However, lower RCCs and radioactive side products were observed when elongating the oxidation reaction time to 20 min (Table 1, entry 8).

**Table 1:** Optimization of the Oxidative Formation of the [^18^F]FPG

entry	oxidation reagent	temperature (°C)	time (min)	RCCs^d^ (%, *n* = 3)
**1**^a^	H_2_SeO_3_ (25 mg, 194 μmol)	110	20	16 ± 1
**2**^a^	H_2_SeO_3_ (50 mg, 388 μmol)	110	20	35 ± 4
**3**^a^	H_2_SeO_3_ (100 mg, 776 μmol)	110	20	60 ± 7
**4**^b^	SeO_2_ (5 mg, 45 μmol)	110	20	56 ± 4
**5**^b^	SeO_2_ (25 mg, 225 μmol)	110	20	84 ± 3
**6**^b^	SeO_2_ (50 mg, 450 μmol)	110	20	97 ± 2
**7**^c^	DMSO/I_2_ (25 mg, 100 μmol)	130	10	92 ± 4 (41 ± 8)^e^
**8**^c^	DMSO/I_2_ (25 mg, 100 μmol)	130	20	86 ± 6

a The purified [^18^F]FAcP was oxidized by H_2_SeO_3_.

b The purified [^18^F]FAcP was oxidized by SeO_2_.

c 4-Acetyl-*N*,*N*,*N*-trimethylbenzenammonium triflate (4.9 mg, 15 μmol) was reacted with [^18^F]KF/K_2.2.2_ in anhydrous DMSO at 110 °C for 10 min. I_2_ (100 μmol) was then added and heated at 130 °C.

d RCCs were determined by HPLC.

e n.d.c. isolated RCYs (*n* = 10).

### Lipophilicity and *In Vitro* Stability of [^18^F]FPG

The lipophilicity of [^18^F]FPG was measured by a conventional partition method between *n*-octanol and phosphate-buffered saline (PBS) at pH 7.4. It has a log *D* of 1.61 ± 0.07 (*n* = 6). The *in vitro* stability of [^18^F]FPG was also determined in 2% DMSO in PBS at 37 °C. No degradation of [^18^F]FPG was observed for up to 4 h (Figure S1).

### Bioconjugation of [^18^F]FPG with HSA

**Figure 2:**
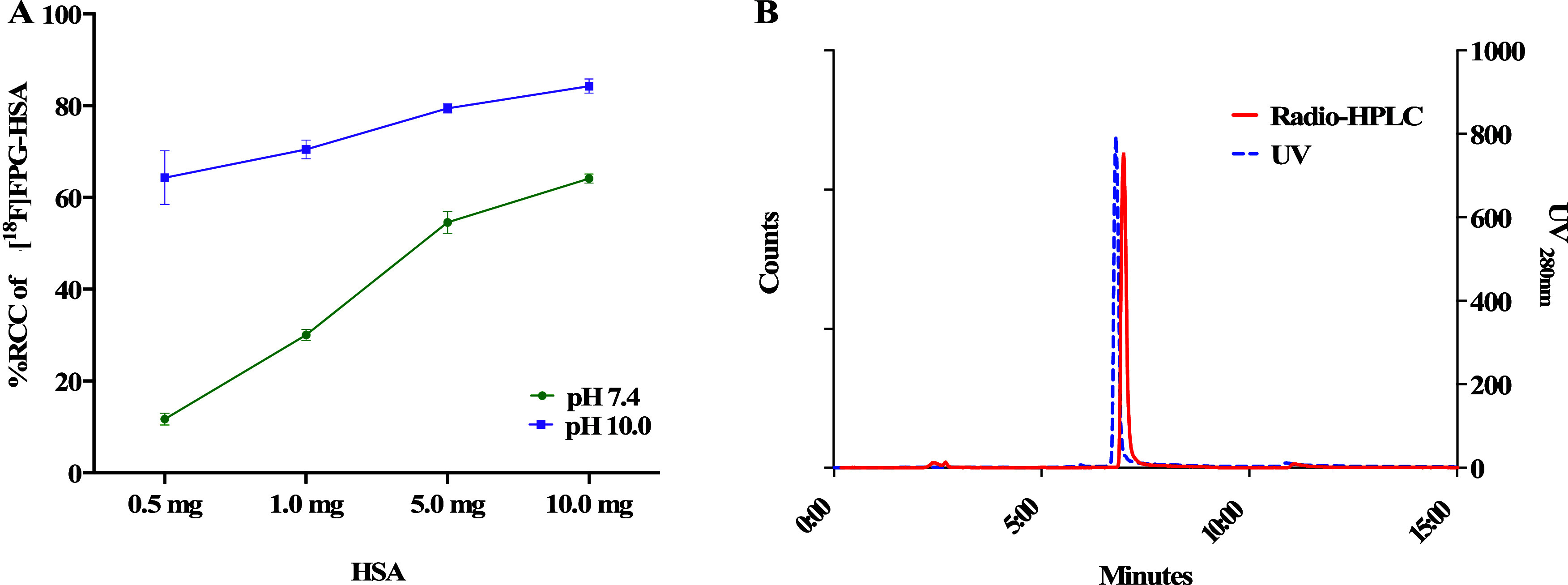
(A) Optimization of [^18^F]FPG bioconjugation with HSA (*n* = 3) and (B) coelution of [^18^F]FPG-HSA with native HSA.

With [^18^F]FPG in hand, we investigated its bioconjugation with HSA (66.5 kDa), which contains 23 arginine residues.^22^ [^18^F]FPG in DMSO (50 μL) was incubated with 0.5–10.0 mg of HSA in either pH 7.4 or pH 10.0 phosphate buffer (450 μL), respectively, at 37 °C for 15 min and then subjected to HPLC analysis. The RCCs from [^18^F]FPG to the [^18^F]FPG-HSA conjugate increased with the increasing HSA loading from 0.5 to 10.0 mg under both pH conditions, as expected (Figure 2A). The RCCs were significantly higher at pH 10.0 than those at pH 7.4. RCCs of 64 ± 6% (*n* = 3) were obtained even at low HSA loading (0.5 mg, 0.75 nmoL) at pH 10.0. After size exclusion purification, the [^18^F]FPG-HSA conjugate was produced in an n.d.c isolated RCY of 61 ± 2% from [^18^F]FPG (*n* = 3). The [^18^F]FPG-HSA coelutes with the native HSA (Figure 2B). The radiochemical purity of [^18^F]FPG-HSA was >98% with a molar activity of 26 ± 6 GBq/μmol (*n* = 3) when starting from 2.2 to 3.9 GBq of [^18^F]FPG.

### Chemoselectivity of [^18^F]FPG Bioconjugation

**Figure 3:**
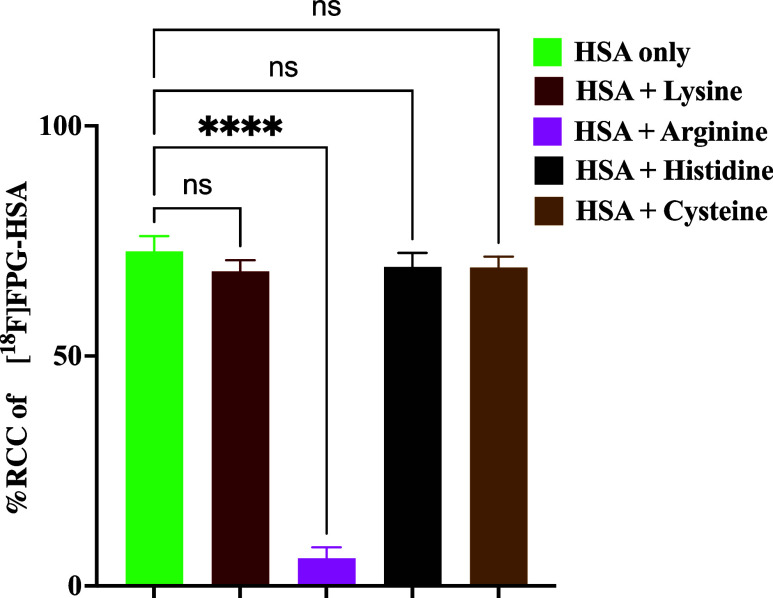
Blocking study of [^18^F]FPG conjugation with HSA using a 30-fold excess of lysine, arginine, histidine, or cysteine (*n* = 3). The control reactions of [^18^F]FPG conjugation with HSA were repeated four times.

In order to determine the chemoselectivity of the [^18^F]FPG bioconjugation, the reaction mixture of HSA and [^18^F]FPG was coincubated with a 30-fold excess of arginine, lysine, histidine, or cysteine representing the corresponding amino acid residues present in HSA.^16^ The reactions were allowed to proceed in pH 10 phosphate buffer at 37 °C for 15 min, followed by radio-HPLC analysis. Control reactions (*n* = 4) were also performed in the absence of each amino acid to compare the RCCs of the [^18^F]FPG-HSA formation. There were no significant differences in the RCCs between the control experiments and those with coincubation with lysine, histidine, or cysteine. In contrast, arginine largely inhibited the formation of [^18^F]FPG-HSA by 93 ± 3% (*n* = 3) (Figure 3).

In addition, we conjugated [^18^F]FPG to both the bovine ubiquitin (8565 Da) and the methylated bovine ubiquitin (8790 Da). These well-characterized proteins contain four arginine residues and seven lysine residues. However, all lysine’s ε-NH_2_ groups of the methylated bovine ubiquitin are methylated (ε-N(CH_3_)_2_), increasing steric hindrance and reducing nucleophilicity of the amine lone pair electrons, potentially eliminating their involvement during bioconjugation with [^18^F]FPG. Similar n.d.c. isolated RCYs of 30 ± 2 and 28 ± 2% (*n* = 2) were obtained for the [^18^F]FPG-ubiquitin and the [^18^F]FPG-methylated ubiquitin, respectively (Figures S2 and S3). These results further illustrate the chemoselectivity of [^18^F]FPG toward arginine residues rather than lysine residues.

To investigate the possible chemical structure of the [^18^F]FPG conjugate with arginine, the nonradioactive reference compound 4-FPG was conjugated with bovine ubiquitin under the conditions used in the radiolabeling. After size exclusion purification, 4-FPG-bovine ubiquitin was analyzed by ESI-MS. The ubiquitin showed a deconvoluted *m*/*z* of 8565.0 (Figure S6A). The 4-FPG-bovine ubiquitin had a deconvoluted *m*/*z* of 8701.1 (Figure S6B). The mass difference between the two proteins is 136.1, which agrees with the mass of either the 4-fluoro-phenyl imidazole-5-ol or its tautomer, 4-fluoro-phenyl imidazolone (Figure S6C).

### *In Vitro* Stability of [^18^F]FPG-HSA

**Figure 4:**
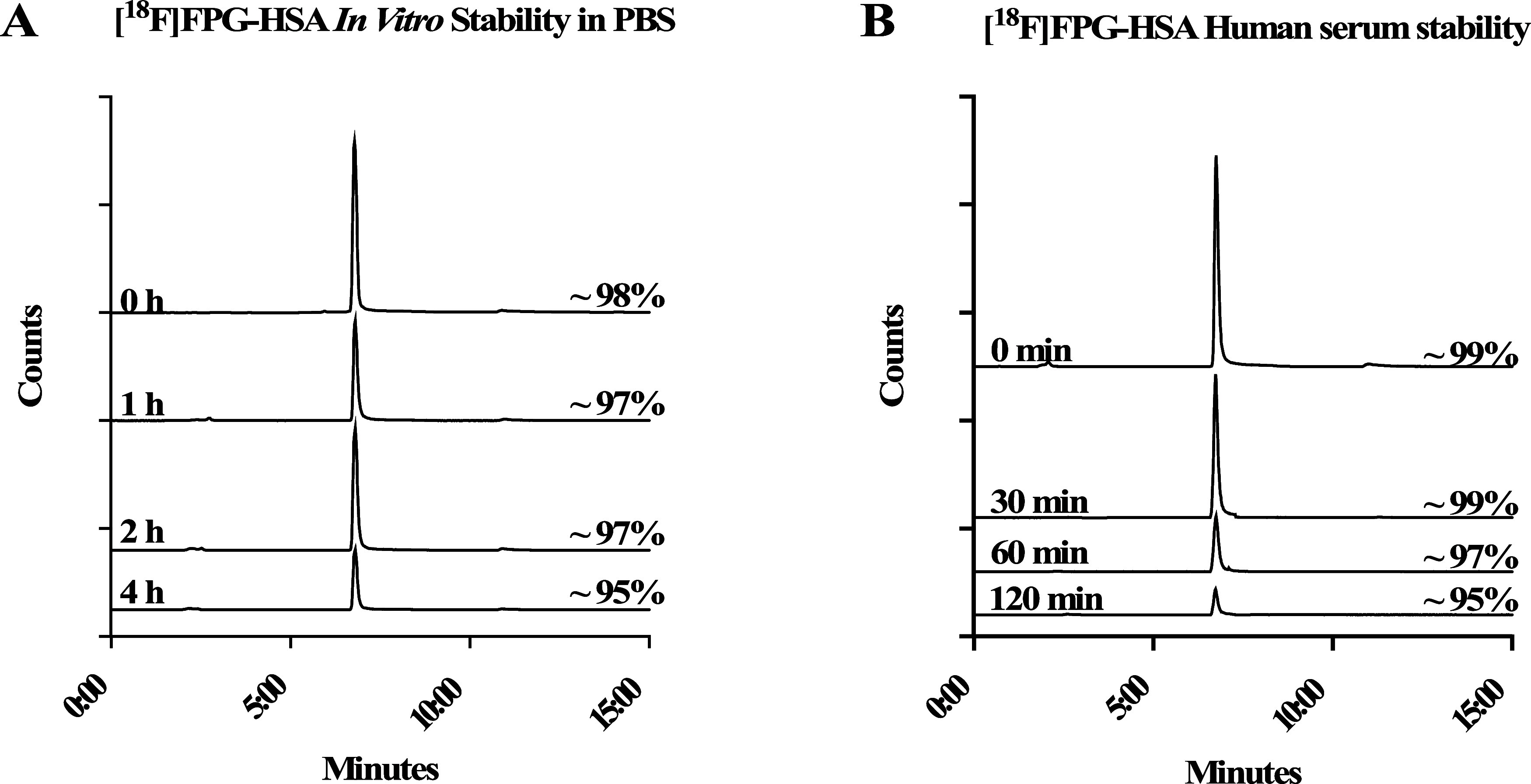
Stability of [^18^F]FPG-HSA at 37 °C determined by radio-HPLC (A) in PBS for 4 h and (B) in human serum for 2 h.

The purified [^18^F]FPG-HSA was incubated at 37 °C in either PBS for 4 h or human serum for 2 h and its stability was monitored via radio-HPLC. In both PBS and human serum, the radiochemical purity of [^18^F]FPG-HSA remained ≥95% over 4 and 2 h, respectively (Figure 4A,B).

### PET Imaging and *Ex Vivo* Biodistribution Studies

**Figure 5:**
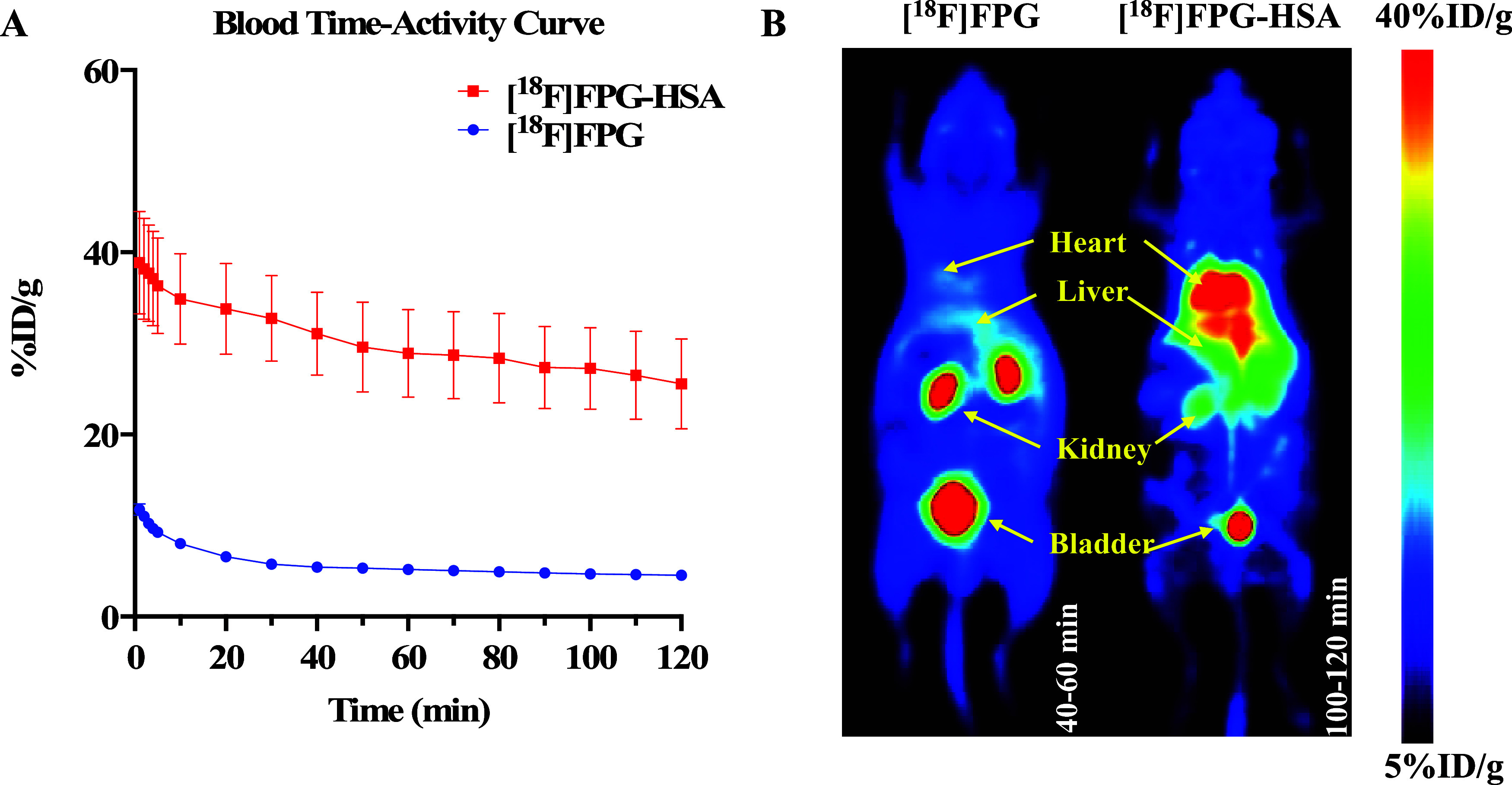
(A) Blood time–activity curve of [^18^F]FPG and [^18^F]FPG-HSA in healthy mice over 120 min (mean ± SD, *n* = 3). (B) The representative maximum intensity projection PET images of [^18^F]FPG (40–60 min) and [^18^F]FPG-HSA (100–120 min) in healthy mice (*n* = 3).

Either [^18^F]FPG (10 ± 5 MBq) or [^18^F]FPG-HSA (9 ± 1 MBq) was administered by tail vein injection in healthy BALB/c mice (*n* = 3, per group), and dynamic PET imaging was performed over 120 min. The blood time–activity curves were measured from the regions of interest (ROIs) in the left ventricular (LV) chamber from the PET image of each animal. The time–activity curves of other major organs are also determined and presented in Figures S7 and S8. The [^18^F]FPG-HSA had significantly higher blood retention compared to [^18^F]FPG throughout the 120 min PET scans. The majority of ^18^F-FPG-HSA was still in circulation 120 min after IV injection (Figure 5A). Additionally, in the 40–60 min PET image of [^18^F]FPG, most of the radioactivity was excreted through the kidney and accumulated in the bladder. By contrast, the [^18^F]FPG-HSA was retained in the blood circulation indicated by the very high LV chamber radioactivity accumulation in the 100–120 min PET image (Figure 5B).

**Figure 6:**
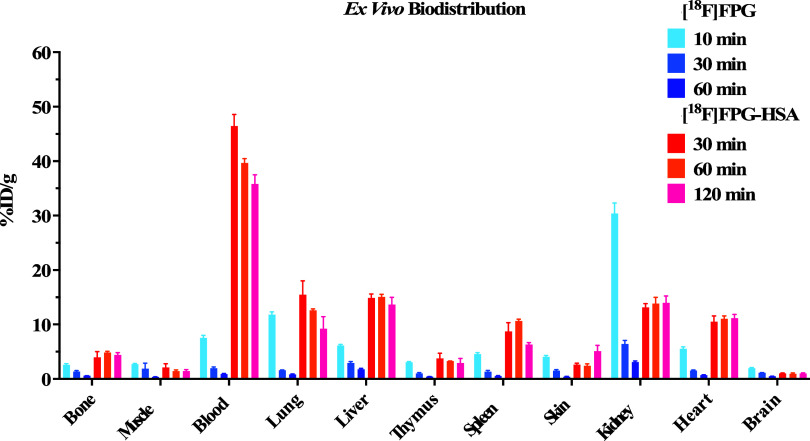
Biodistribution of [^18^F]FPG at 10, 30, or 60 min and [^18^F]FPG-HSA at 30, 60, or 120 min post IV injection (mean ± SD, *n* = 3, per time point).

The *ex vivo* biodistribution study using healthy BALB/c mice was conducted with [^18^F]FPG (∼1 MBq) at 10, 30, or 60 min post IV injection and [^18^F]FPG-HSA (∼1 MBq) at 30, 60, or 120 min post IV injection (*n* = 3, per time point) (Figure 6). [^18^F]FPG was rapidly cleared from the blood and all other major organs, and only 1 ± 0.2% ID/g of radioactivity was present in blood 60 min postinjection. While 36 ± 3% ID/g of radioactivity remained in blood from the group injected with [^18^F]FPG-HSA 120 min postinjection (Table S1). The radioactivity in the lung, liver, spleen, kidney, and heart remained largely constant between 10 and 20% ID/g at all three time points in the animals that received the [^18^F]FPG-HSA. The bone uptakes for [^18^F]FPG and [^18^F]FPG-HSA were 0.6 ± 0.1% ID/g at 60 min post IV injection and 4.4 ± 0.7% ID/g at 120 min post IV injection, respectively (Table S1).

### Bioconjugation of [^18^F]FPG with Human Interleukin-2 and Interleukin-4

We investigated whether proteins labeled with [^18^F]FPG at their arginine residues retain the binding affinity of their respective nonconjugated native proteins. [^18^F]FPG (∼20 MBq) was conjugated to human interleukin-2 (IL-2, 15.3 kDa) and human interleukin-4 (IL-4, 15.1 kDa) in pH 10 phosphate buffer at 37 °C for 15 min and purified by size exclusion. The n.d.c. isolated RCY of [^18^F]FPG-IL-2 was 31 ± 2% (*n* = 3) with a radiochemical purity >96% (Figure S4). The n.d.c. isolated RCY of [^18^F]FPG-IL-4 was 28 ± 3% (*n* = 3) with a radiochemical purity >98% (Figure S5).

### Binding Affinity of [^18^F]FPG-IL-2 and [^18^F]FPG-IL-4

**Figure 7:**
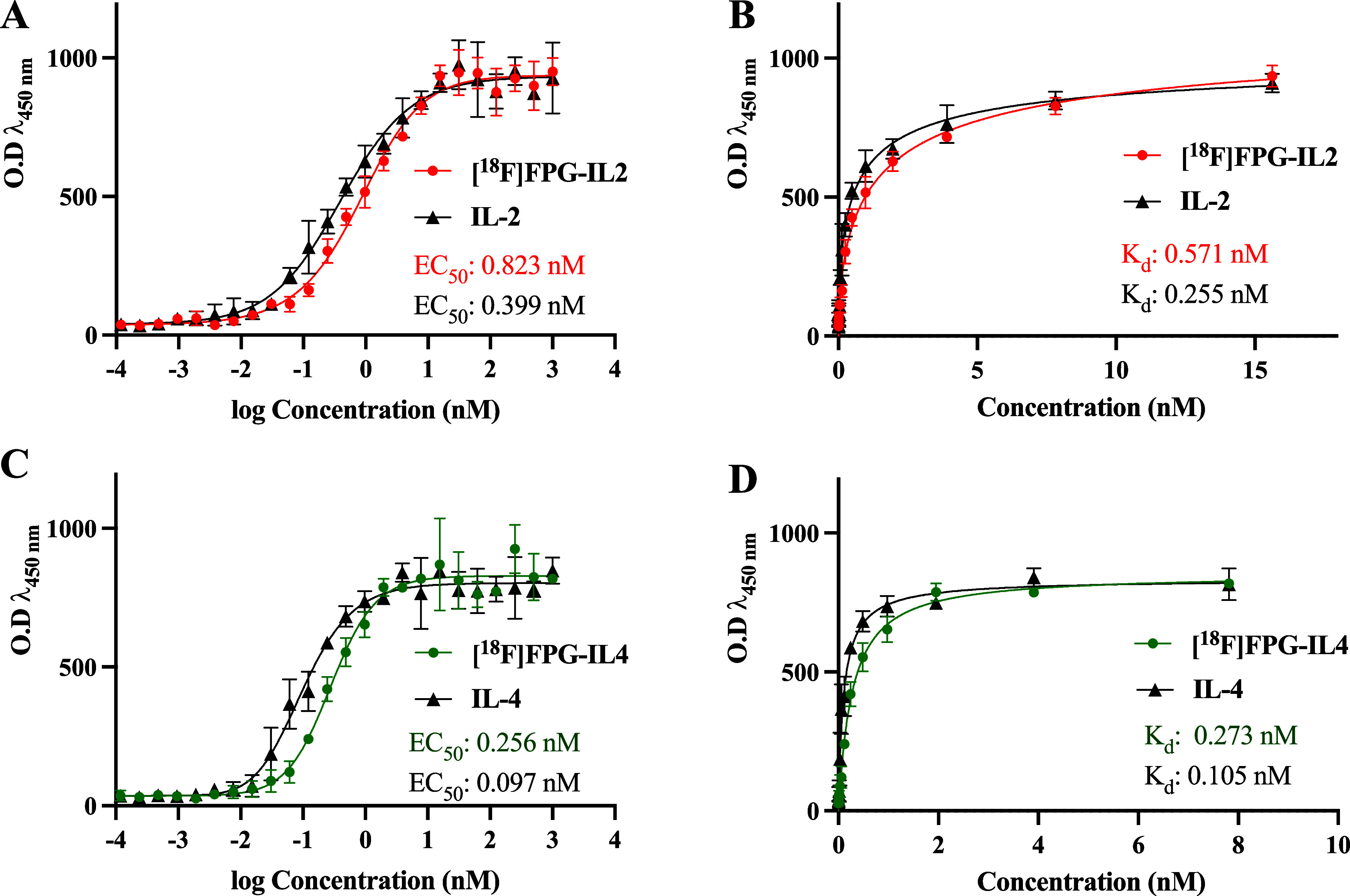
(A) EC_50_ of [^18^F]FPG-IL-2 and IL-2, (B) *K*_d_ of [^18^F]FPG-IL-2 and IL-2, (C) EC_50_ of [^18^F]FPG-IL-4 and IL-4, and (D) *K*_d_ of [^18^F]FPG-IL-4 and IL-4 (*n* = 3).

The EC_50_ and *K*_d_ values of [^18^F]FPG-IL-2, native IL-2, [^18^F]FPG-IL-4, and native IL-4 were determined using enzyme-linked immunosorbent assays (ELISAs). The EC_50_ values of [^18^F]FPG-IL-2 and native IL-2 were 0.823 nM and 0.399 nM, respectively (Figure 7A). The *K*_d_ values for [^18^F]FPG-IL-2 and native IL-2 were 0.571 nM and 0.255 nM, respectively (Figure 7B). Similarly, the EC_50_ values of the conjugate [^18^F]FPG-IL-4 and the native IL-4 were 0.256 nM and 0.097 nM, respectively (Figure 7C), with their respective *K*_d_ values being 0.273 nM for [^18^F]FPG-IL-4 and 0.105 nM for the native IL-4 protein (Figure 7D). In all cases, conjugation with [^18^F]FPG had a minimal impact on either the EC_50_ or *K*_d_ conjugates versus the native proteins.

## Discussion

To develop a late-stage ^18^F-labeling method for [^18^F]FPG radiosynthesis, we attempted the direct fluorination of 4-chlorophenylglyoxal and 4-nitrophenylglyoxal with fluoride-18. However, the formation of [^18^F]FPG was not observed (data not shown). As the glyoxal is in equilibrium with its hydrate form, the labile hydroxyl protons inhibit the reactivity of fluoride-18 through hydrogen bonding. An alternative one-pot, two-step radiosynthesis strategy to prepare the [^18^F]FPG via oxidation of the [^18^F]FAcP intermediate was investigated. [^18^F]FAcP was prepared by the S_N_Ar [^18^F]fluorination of 4-acetyl-*N*,*N*,*N*-trimethylbenzenammonium triflate precursor in anhydrous DMSO. Despite the quantitative conversion of fluoride-18 to [^18^F]FAcP was observed by HPLC, we experienced a significant loss of [^18^F]FAcP during the purification processes, resulting in lower isolated RCYs of ∼30%. Nevertheless, sufficient [^18^F]FAcP was obtained to investigate the selenium-based Riley oxidation to convert [^18^F]FAcP to [^18^F]FPG. SeO_2_ exhibited superior reactivity compared to H_2_SeO_3_ and provided 97 ± 2% RCCs by oxidizing purified [^18^F]FAcP to [^18^F]FPG in 1,4-dioxane/H_2_O solution. However, attempts to convert this method into a one-pot process failed to produce [^18^F]FPG from the crude reaction mixture of [^18^F]FAcP. Potential causes can be attributed to the interference of SeO_2_ with K_2_CO_3_ and the unreacted precursor as well as its reduced solubility in anhydrous DMSO. Fortunately, the I_2_-mediated Kornblum oxidation using DMSO as an oxidant was successful. [^18^F]FPG was obtained in ∼41% n.d.c. isolated RCYs within ∼60 min in a one-pot, two-step radiosynthesis starting from fluoride-18.

Human serum albumin (HSA) was selected as a reference protein to demonstrate [^18^F]FPG bioconjugation and chemoselectivity, as it is well-characterized and readily available. The resultant [^18^F]FPG-HSA conjugate has potential applications as a blood pool PET imaging agent.^23,24^ [^18^F]FPG effectively conjugated to HSA at pH 10.0. The % RCCs were significantly lower at pH 7.4 [i.e., 0.5 mg of HSA has 12 ± 1% RCCs at pH 7.4 compared to 64 ± 6% RCCs at pH 9–10 (*n* = 3)]. The p*K*_a_ of the guanidinium group in arginine is 13.8 and is positively charged at pH 7.4.^25^ This reduces its nucleophilicity toward the glyoxal group in the [^18^F]FPG, resulting in lower % RCC at lower pH. Traditionally, bioconjugation of lysine residues with [^18^F]SFB required pH 8.0–8.5 and elevated temperatures for acceptable radiochemical yields.^29^ More recently, generational analogues of [^18^F]SFB, such as [^18^F]TFPFN, have improved the radiosynthesis of labeled proteins but still required high masses of protein for bioconjugation (20 mg of rat serum albumin to achieve “∼18–35% uncorrected yield”, pH 9 buffer at 40 °C for 15 min).^6^ Other prosthetic groups rely on protein modification prior to bioconjugation. For instance, [^18^F]FBA was employed to label hydrazinonicotinic acid (HYNIC)-modified HSA.^12^ The conjugation required elevated temperatures of 60 °C, a high degree of protein modification, and increased protein mass (3 mg) to afford [^18^F]fluorobenzyl-HSA in ∼80% RCC. In comparison, [^18^F]FPG required just 0.5 mg of HSA to achieve effective radiolabeling in an n.d.c isolated RCY of 61 ± 2% at 37 °C without the need for protein modification. The ability to radiolabel proteins at relatively low substrate concentrations and under mild bioconjugation conditions indicates that [^18^F]FPG may well be suitable for the economical radiolabeling of precious and heat-sensitive proteins.

The chemoselectivity of [^18^F]FPG was evaluated by reacting it with HSA coincubated with a 30-fold molar excess of arginine, lysine, cysteine, or histidine. Only arginine significantly inhibited the bioconjugation of [^18^F]FPG with HSA compared with the other amino acids. The chemoselectivity of [^18^F]FPG toward arginine was further evaluated through bioconjugation with both bovine ubiquitin and the corresponding lysine methylated bovine ubiquitin. Almost identical isolated RCYs of ∼30% were obtained from both experiments, providing further evidence that [^18^F]FPG is arginine-selective. The chemoselectivity of glyoxals toward arginine has been attributed to the formation of thermodynamically stable cyclic products.^15^ In contrast, the hemithioacetal and imine products formed between the condensation of glyoxals with cysteine and lysine, respectively, can rapidly be hydrolyzed under physiological conditions.^26,27^ Indeed, [^18^F]FPG-HSA exhibits excellent stability under physiological conditions. Furthermore, the mass spectrometry data of the 4-FPG conjugated bovine ubiquitin and native bovine ubiquitin afforded a *m*/*z* difference of 136.1. The fragment indicates the chemical structure of the 4-FPG and arginine conjugate could be either the 4-fluoro-phenyl imidazole-5-ol or its tautomer, 4-fluoro-phenyl imidazolone, analogous to literature reports.^16^

To demonstrate potential applications of the [^18^F]FPG-labeled proteins as PET imaging reagents, [^18^F]FPG-HSA was evaluated in healthy animals. Both the PET imaging and the *ex vivo* biodistribution studies have shown prolonged circulation time of [^18^F]FPG-HSA in blood, illustrating its potential as a blood pool PET imaging agent. Moreover, the biodistribution profile of [^18^F]FPG-HSA is in good agreement with [^18^F]FBA-labeled HSA apart from the bone uptakes reported by Lee.^12^ The bone uptakes from the mice that received [^18^F]FPG-HSA were significantly lower (4–5%) and at much later time points (30 to 120 min) than those of [^18^F]FBA-labeled HSA (8–10% from 10 to 60 min), which indicates that [^18^F]FPG-HSA is more resistant toward *in vivo* defluorination than [^18^F]FBA-labeled HSA. Additionally, the constant bone uptakes (∼4% ID/g in 120 min) of [^18^F]FPG-HSA are likely due to the very high blood retention of [^18^F]FPG-HSA (>36% ID/g at 120 min postinjection) rather than its defluorination *in vivo*. Moreover, the bone uptakes of [^18^F]FPG were also at a low level of <3% ID/g and rapidly decreased from ∼2.6 to ∼0.6% ID/g in 60 min post IV injections. All of these data give us confidence that both [^18^F]FPG and its protein conjugates are resistant toward *in vivo* defluorination.

Furthermore, Wuest has reported the use of [^18^F]fluoro-*N*-methyl-*N*-(prop-2-ynyl)-benzenesulfonamide (*p*[^18^F]F-SA) to label azide-modified HSA via copper-mediated click chemistry.^30^ However, the *p*[^18^F]F-SA-labeled HSA showed fast blood clearance in healthy mice PET imaging. The authors claimed that both the azide modification and the click chemistry have significantly altered the structural and functional integrity of HSA. In contrast, [^18^F]FPG-HSA has shown prolonged blood retention in both PET imaging and biodistribution, which indicates that [^18^F]FPG-based bioconjugation preserves the biological properties of HSA. Thus, both the PET imaging and the *ex vivo* biodistribution data of [^18^F]FPG-HSA provide convincing evidence that the imidazole-5-ol or imidazolone moiety formed in the [^18^F]FPG-labeled proteins has excellent biostability *in vivo*. In clinical practice, the technetium-99m-labeled HSA (^99m^Tc-HSA) is the main radiopharmaceutical for the blood pool SPECT imaging to investigate cardiac function and detect infection and gastrointestinal bleeding.^28^ [^18^F]FPG-HSA could serve as a PET imaging agent that provides improved image resolution due to the intrinsic higher resolution of PET.

A critical characteristic of a bioconjugation reagent is to preserve the binding affinity of the native proteins. Given the low molecular weight of the adduct, it was predicted that the pharmacokinetics and pharmacodynamics of the modified protein should not be altered. Thus, we radiolabeled two cytokines, IL-2 and IL-4, with [^18^F]FPG, with the intent of tracking T-cells overexpressing IL-2/IL-4 receptors in preclinical oncology models. [^18^F]FPG-IL-2 was obtained in 31 ± 2% (*n* = 3) RCYs, which is significantly higher than [^18^F]SFB-labeled IL-2 (∼19%).^29^ While [^18^F]FPG-IL-4 was obtained in 28 ± 2% (*n* = 3) RCYs. To the best of our knowledge, the binding affinity of radiolabeled IL-2 has not been reported, and this is the first report of radiolabeled IL-4. We then decided to use the standard ELISA methods to determine the binding affinity (EC_50_ and *K*_d_) of [^18^F]FPG-IL-2 and [^18^F]FPG-IL-4 as well as native IL-2 and IL-4 for comparison. Comparable EC_50_ and *K*_d_ values of [^18^F]FPG-IL-2 and IL-2 as well as [^18^F]FPG-IL-4 and IL-4 in the subnanomolar range were observed. The data indicate that [^18^F]FPG-labeled IL-2 and IL-4 retain the binding affinity of their corresponding native proteins. It is promising to note that the affinity of [^18^F]FPG-IL-2 and [^18^F]FPG-IL-4 toward IL-2 and IL-4 receptors is retained and it is anticipated that their behavior *in vivo* will be similar to that of the parent proteins. Currently, we are investigating applications of both [^18^F]FPG-IL-2 and [^18^F]FPG-IL-4 for the *in vivo* tracking of CAR-T cells. However, as a novel prosthetic group, the versatility of [^18^F]FPG-based bioconjugation remains to be thoroughly examined, particularly for proteins sensitive to elevated pH levels.

## Conclusions

A novel bioconjugation reagent, [^18^F]FPG, has been radiosynthesized in a one-pot, two-step process with good radiochemical yield and high radiochemical purity. [^18^F]FPG effectively radiolabels five proteins, including HSA, bovine ubiquitin, methylated bovine ubiquitin, IL-2, and IL-4 through selective bioconjugation of arginine residues. The selectivity for arginine functional groups is evidenced by the inhibition of [^18^F]FPG conjugation with HSA in the presence of excess arginine, whereas in the presence of other nucleophilic amino acids, such as lysine, cysteine, and histidine, bioconjugation was unimpeded. Mass spectrometry studies of the 4-FPG-bovine ubiquitin indicated that 4-FPG couples with arginine to form either a 4-fluoro-phenyl imidazole-5-ol or its tautomer, 4-fluoro-phenyl imidazolone. The [^18^F]FPG-HSA conjugate has excellent *in vivo* stability and long circulation time, which demonstrates that [^18^F]FPG-HSA has potential as a blood pool contrast reagent for PET imaging. Thus, [^18^F]FPG is a robust and selective prosthetic group for developing protein-based ^18^F-PET imaging agents.

## Experimental Section

### General Information

^1^H and ^13^C NMR spectra were recorded at room temperature (RT) on a Bruker Avance 400 instrument operating at a frequency of 400 MHz for ^1^H, 100 MHz for ^13^C, and 376 MHz for ^19^F (United Kingdom). Chemical shifts are reported in ppm relative to DMSO-*d*_6_ (δ 2.48, m) or CDCl_3_ (δ 7.26, s), and coupling constants (*J*) are given in Hertz. Radio-HPLC analysis was performed with an Agilent 1200 HPLC system equipped with a 1200 series diode array detector and Raytest GABI Star radioactivity detector (United Kingdom) or UFLC Shimadzu radio-HPLC systems (Shimadzu, Singapore). Radioactivity measurements were recorded with a CRC-55tPET dose calibrator (Capintec, Florham Park, NJ). All of the reagents were purchased from Sigma-Aldrich, while 4-fluorophenylglyoxal was purchased from FischerScientific. All of the commercially available chemicals were used without purification. Sep-Pak light (46 mg) Accell, QMA carbonate, and Oasis HLB cartridges were purchased from Waters Pacific Pte Ltd., Singapore. PD-10 desalting columns were obtained from GE Healthcare Life Sciences, Singapore. Human serum albumin was purchased from Sigma-Aldrich. Methylated ubiquitin was purchased from Enzo Life Sciences, Inc., Switzerland. Proleukin (aldesleukin, 18 × 10^6^ IU), a recombinant interleukin-2 (desalanyl-1, serine-125 human interleukin-2), was acquired from Novartis, Singapore. Human interleukin-4 was acquired from GenScript Biotech, United Kingdom. No-carrier-added (n.c.a.) aqueous fluoride-18 was produced by the irradiation of ^18^O-enriched water via the ^18^O(*p*, *n*)^18^F nuclear reaction using a GE PETtrace 860 cyclotron at either the PET Center at St. Thomas’ Hospital (United Kingdom) or Clinical Research Imaging Centre (National University of Singapore). All compounds are >95% pure by HPLC analysis.

### Organic Synthesis

#### Synthesis of 4-Acetyl-*N*,*N*,*N*-trimethylbenzenammonium Triflate^18^

1-(4-(Dimethylamino)phenyl)ethan-1-one (245 mg, 1.5 mmol) was dissolved in Et_2_O (10 mL) in a 50 mL round-bottom flask containing a magnetic stir bar, and the solution was cooled to 0 °C. Methyl trifluoromethanesulfonate (0.35 mL, 3.0 mmol) was added dropwise to the stirred solution and allowed to warm up to room temperature over 2 h. The precipitate was collected and washed twice with ice-cold Et_2_O to produce the title product as a white solid (410 mg, 83%). ^1^H NMR (400 MHz, DMSO-*d*_6_) δ 8.11–8.18 (4H, m), 3.65 (9H, s), 2.66 (3H, s). ^13^C NMR (100 MHz, DMSO-*d*_6_) δ: 197.50, 150.70, 138.22, 130.25, 121.62, 56.85, 27.54. ^19^F NMR (376 MHz, DMSO-*d*_6_) δ −77.74. The NMR data are in agreement with the literature data.^18^

### Radiosynthesis

#### General Procedure for Azeotropic Distillation

Fluoride-18 (7-10 GBq) was trapped onto a preactivated QMA-light cartridge and released with 1.0 mL of K_2.2.2_/K_2_CO_3_ (30/15 mM) in acetonitrile/water (85/15) in a 5 mL Wheaton vial. The solvent was evaporated under a stream of N_2_ (500 cm^3^/s) at 110 °C under vacuum (400 mbar) for 5 min. After complete removal of solvents, azeotropic distillation with anhydrous acetonitrile (0.5 mL) was repeated twice at 110 °C for 10 min.

#### 4-[^18^F]Fluoroacetophenone

4-Acetyl-*N*,*N*,*N*-trimethylbenzenammonium triflate (4.9 mg, 15 μmol) was dissolved in anhydrous DMSO (500 μL) and added to the [^18^F]KF/K_2.2.2_ complex and heated at 110 °C for 5 min. The reaction mixture was diluted with 25 mL of H_2_O and passed through an HLB Plus cartridge (preconditioned with 2 mL of 1,4-dioxane followed by 20 mL of H_2_O) and the cartridge was eluted with 1,4-dioxane/H_2_O (20:1, 500 μL) to afford the product in an n.d.c. RCY of 30 ± 8% (*n* = 6), over an average synthesis duration of 59 ± 11 min (*n* = 6).

#### 4-[^18^F]Fluorophenylglyoxal via H_2_SeO_3_ Oxidation

An aliquot of 4-[^18^F]fluoroacetophenone (50–100 MBq) in 1,4-dioxane/water solution (500 μL, 20:1) was transferred to a 5 mL Wheaton vial, to which 25 mg, 50 mg, or 100 mg of H_2_SeO_3_ in H_2_O (25 μL) was added. The reaction mixture was heated at 110 °C for 20 min. The reaction was quenched with H_2_O (1.5 mL) and then analyzed via radio-HPLC. Luna 5 μm Phenyl-Hexyl 100 Å, LC column 250 × 4.6 mm; mobile phase: 0.1% trifluoroacetic acid in both MeCN and H_2_O. Method: 25–95% MeCN from 0 to 9 min; 95% MeCN from 9 to 10 min; 95–5% MeCN from 10 to 12 min. Flow rate: 1.0 mL/min.

#### 4-[^18^F]Fluorophenylglyoxal via SeO_2_ Oxidation

An aliquot of [^18^F]fluoroacetophenone (50–100 MBq) was eluted with 1,4-dioxane/H_2_O (500 μL, 20:1) directly into a 5 mL Wheaton vial containing 5 mg, 25 mg, or 50 mg of SeO_2_. The reaction was heated at 110 °C for 20 min and then diluted with 1.5 mL of H_2_O and analyzed via HPLC. Semipreparative HPLC: Agilent column, XDB-C18, semipreparative, 100 Å, 9.4 × 250 mm, 5 μm. Mobile phase: 0.1% trifluoroacetic acid in both MeCN and H_2_O. Method: 5–95% MeCN from 0 to 15 min; 95% MeCN from 15 to 18 min; 95–5% MeCN from 18 to 20 min. Flow rate: 3.0 mL/min.

#### One-Pot, Two-Step Radiosynthesis of 4-[^18^F]fluorophenylglyoxal

4-Acetyl-*N*,*N*,*N*-trimethylbenzenammonium triflate (4.9 mg, 15 μmol) was dissolved in anhydrous DMSO (500 μL) and added to the [^18^F]KF/K_2.2.2_ complex and heated at 110 °C for 10 min. I_2_ (25 mg, 100 μmol) was then added to the reaction mixture and heated at 130 °C for another 10 min. The reaction was quenched with a saturated Na_2_S_2_O_3_ solution (25–150 μL) and purified via semipreparative HPLC. Semipreparative HPLC: Prodigy 5 μm ODS-3 100 Å LC Column 250 × 10 mm. Mobile phase: 0.1% trifluoroacetic acid in both MeCN and H_2_O. Method: 5% MeCN from 0 to 5 min; 40–100% MeCN from 5 to 15 min; 100% MeCN from 15 to 20 min; 100–5% MeCN from 20 to 25 min. Flow rate = 3.0 mL/min. The product fraction (*t*_R_ = 15.30 min) was collected, diluted, and trapped in an OASIS HLB cartridge, and the cartridge was eluted with DMSO. [^18^F]FPG was obtained in an n.d.c. isolated RCY of 41 ± 8% with RCP > 99%, with a molar activity of 303 ± 104 GBq/μmol, over 56 ± 6 min (*n* = 10). A coelution of the [^18^F]FPG with its nonradioactive reference compound (*t*_R_ = 6.23 min) was performed to confirm the identity of the radioactive compound (*t*_R_ = 6.25 min). Analytical HPLC: Aeris 5 μm Peptide XB-C18. 250 × 4.6 mm Mobile phase: 0.1% trifluoroacetic acid in both MeCN and H_2_O. Method: 5–95% MeCN from 0 to 5 min; 95% MeCN for 5–6 min; 95–5% MeCN for 6–12 min. Flow rate = 1.0 mL/min. [^18^F]FPG was formulated in 2% DMSO in pH 7.4 PBS for preclinical PET imaging.

### Log *D* Measurement

The log *D* of [^18^F]FPG was determined via the partition method. *n*-Octanol was presaturated with PBS (pH 7.4) before use. [^18^F]FPG (∼0.2 MBq) was added to a mixture of PBS (500 μL) and *n*-octanol (500 μL) in a 1.5 mL Eppendorf vial (*n* = 6). The mixture was vigorously agitated under ambient conditions and then centrifuged at 5000 rpm for 5 min. A 100 μL aliquot from each layer was drawn for measurement on a γ counter. The log *D*_oct/PBS_ was calculated as follows: log [(cpm in the 1-octanol layer – cpm 1-octanol blank)/(cpm in the PBS layer – cpm PBS blank)].

### Bioconjugation

#### Optimization of [^18^F]FPG Conjugation with HSA

[^18^F]FPG (20–42 MBq) in DMSO (50 μL) was added to human serum albumin (10.00 5.00, 1.00, or 0.50 mg) in pH 7.4 PBS (450 μL). The reaction mixture was adjusted to pH 10.0 with NEt_3_ and incubated at 37 °C for 15 min. The above reactions were repeated in pH 7.4 PBS at 37 °C for 15 min. All reactions were analyzed using radio-HPLC. Analytical HPLC: Jupiter 5 μm C4 300 Å 150 × 4.6 mm Mobile phase: 0.1% trifluoroacetic acid in both MeCN and H_2_O. Method: 5–95% MeCN from 0 to 5 min; 95% MeCN for 5–6 min; 95–5% MeCN for 6 to 14 min. Flow rate = 1.0 mL/min. For preclinical PET imaging: [^18^F]FPG (2.2–3.9 GBq) was added to HSA (1.0 mg, 15 nmol) in pH 7.4 PBS (400 μL). The reaction mixture was adjusted to pH 10.0 with NEt_3_ and incubated at 37 °C for 15 min. The [^18^F]FPG-HSA was purified via a PD-10 column eluting with PBS. A coelution of [^18^F]FPG-HSA with native HSA (*t*_R_ = 7.02 min) was performed to confirm the identity of the radioactive molecule (*t*_R_ = 6.81 min). Analytical HPLC: Phenomenex Aeris Widepore 3.6 μm C4 300 Å 150 × 2.1 mm. Mobile phase: 0.1% trifluoroacetic acid in both MeCN and H_2_O. Method: 5–95% MeCN from 0 to 5 min; 95% MeCN for 5–6 min; 95–5% MeCN for 6–15 min. Flow rate = 1.0 mL/min.

The [^18^F]FPG-HSA was obtained in an n.d.c isolated RCY of 61 ± 2% from [^18^F]FPG in 28 ± 2 min (*n* = 3). The RCP of [^18^F]FPG-HSA was >99% with a molar activity of 26 ± 6 GBq/μmol (*n* = 3). The [^18^F]FPG-HSA was formulated in pH 7.4 PBS for preclinical imaging.

#### Determining the Chemoselectivity of [^18^F]FPG toward Arginine Residues

HSA (15 nmoL) in PBS (450 μL) was mixed with a 30-fold excess of arginine, lysine, histidine, or cysteine to the number of corresponding amino acid residues present in HSA.^22^ The pH was adjusted to ∼10.0 with NEt_3_. [^18^F]FPG (20–42 MBq) in DMSO (50 μL) was then added, and the reaction mixture was incubated at 37 °C for 15 min. Control reactions in the absence of added amino acids were also performed (*n* = 4). All of the reactions were analyzed by radio-HPLC. Analytical HPLC: Jupiter 5 μm C4 300 Å 150 × 4.6 mm. Mobile phase: 0.1% trifluoroacetic acid in both MeCN and H_2_O. Method: 5–95% MeCN from 0 to 5 min; 95% MeCN from 5 to 6 min; 95–5% MeCN from 6 to 15 min. Flow rate = 1.0 mL/min.

#### Bioconjugation of FPG with Bovine Ubiquitin

FPG (23 nmol) in DMSO (3.5 μL) was added to bovine ubiquitin (230 nmol) in PBS (450 μL). The reaction mixture was adjusted to pH ∼10.0 with NEt_3_ and incubated at 37 °C for 15 min. The crude reaction mixture was purified via a PD-10 column collecting 200 μL fractions, which were analyzed by liquid chromatography–mass spectrometry (LC–MS).

### General Procedure of [^18^F]FPG Bioconjugation with Proteins

[^18^F]FPG (20–42 MBq) in DMSO (25 μL) was added to bovine ubiquitin (23 nmol), methylated bovine ubiquitin (23 nmol), human IL-2 (13 nmol), or human IL-4 (13 nmol) in pH 7.4 PBS (225 μL). The reaction mixture was adjusted to pH ∼10.0 with NEt_3_ and incubated at 37 °C for 15 min. The crude reaction mixture was purified via a PD-10 column in 500 μL fractions and analyzed via radio-HPLC. Analytical HPLC: Jupiter 5 μm C4 300 Å 150 × 4.6 mm. Mobile phase: 0.1% trifluoroacetic acid in both MeCN and H_2_O. Method: 5–95% MeCN from 0 to 5 min; 95% MeCN from 5 to 6 min; 95–5% MeCN from 6 to 15 min. Flow rate = 1.0 mL/min.

The [^18^F]FPG-bovine ubiquitin has an HPLC retention time of 5.16 min and was obtained in an n.d.c isolated RCY of 30 ± 2% from [^18^F]FPG in 24 ± 4 min (*n* = 2). The RCP was >99% with a molar activity of 0.5 ± 0.3 GBq/μmol (*n* = 2).

The [^18^F]FPG-methylated bovine ubiquitin has an HPLC retention time of 5.55 min and was obtained in an n.d.c isolated RCY of 28 ± 2% from [^18^F]FPG in 26 ± 2 min (*n* = 2). The RCP was >97% with a molar activity of 0.3 ± 0.1 GBq/μmol (*n* = 2).

The [^18^F]FPG-IL-2 has an HPLC retention time of 7.65 min and was obtained in an n.d.c isolated RCY of 31 ± 2% from [^18^F]FPG in 25 ± 3 min (*n* = 2). The RCP was >96% with a molar activity of 0.6 ± 0.1 GBq/μmol (*n* = 2).

The [^18^F]FPG-IL-4 has an HPLC retention time of 5.57 min and was obtained in an n.d.c isolated RCY of 28 ± 3% from [^18^F]FPG in 27 ± 1 min (*n* = 2). The RCP was >98% with a molar activity of 0.4 ± 0.03 GBq/μmol (*n* = 2).

### *In Vitro* Biological Evaluation of [^18^F]FPG-Conjugated Proteins

#### [^18^F]FPG-HSA Stability in PBS

[^18^F]FPG-HSA (∼40 MBq) in pH 7.4 PBS (500 μL) was incubated at 37 °C and aliquots (25 μL) were taken at 0, 1, 2, and 4 h and analyzed via radio-HPLC. Analytical HPLC: Phenomenex Aeris Widepore 3.6 μm C4 300 Å 150 × 2.1 mm. Mobile phase: 0.1% trifluoroacetic acid in both MeCN and H_2_O. Method: 5–95% MeCN from 0 to 5 min; 95% MeCN for 5–6 min; 95–5% MeCN for 6–15 min. Flow rate = 1.0 mL/min.

#### [^18^F]FPG-HSA Stability in Human Serum

[^18^F]FPG-HSA (∼15 MBq) in PBS (50 μL) was added to human serum (500 μL) and incubated at 37 °C. Aliquots (25 μL) were taken at 0, 30, 60, and 120 min and analyzed directly via radio-HPLC. Analytical HPLC: Phenomenex Aeris Widepore 3.6 μm, C4 300 Å 150 × 2.1 mm. Mobile phase: 0.1% trifluoroacetic acid in both MeCN and H_2_O. Method: 5–95% MeCN from 0 to 5 min; 95% MeCN for 5–6 min; 95–5% MeCN for 6–15 min. Flow rate = 1.0 mL/min.

#### ELISAs of [^18^F]FPG-IL-2 or [^18^F]FPG-IL-4

ELISAs were conducted using Invitrogen human IL-2 and IL-4 uncoated ELISA Kits according to the manufacturer’s instructions. A 200 ng/well antihuman IL-2 or IL-4 capture antibody was coated onto the high binding affinity 96-well ELISA plates in PBS and incubated at 4 °C overnight. The plates were washed 4× with PBS-Tween (0.1%) and the wells were blocked with 1% bovine serum albumin (BSA) at RT for 1 h. The plates were washed again 4× with PBS-Tween (0.1%). Starting with 1000 nM followed by twenty-three 1:2 dilutions of [^18^F]FPG-IL-2, native IL-2, [^18^F]FPG-IL-4, and native IL-4, respectively, were added and incubated at RT for 2 h. The detection antibody (1:250) was then added and the mixture was incubated at RT for 1 h, followed by the addition of Avidin-HRP (1:250) and incubation at RT for another 30 min. The plates were then washed with PBS-Tween (0.1%), and 100 μL of 3,3′,5,5′-tetramethylbenzidine (TMB) was added. The plates were covered with aluminum foil to protect them from light and incubated at RT for 10 min. H_2_SO_4_ (100 μL 2.0 M) was then added to stop the reaction and plates were read on a Biotrack II visible plate reader at an absorbance of 450 nm. Data were plotted and analyzed in GraphPad Prism by nonlinear regression in the logarithmic (agonist) vs response model.

#### *In Vivo* and *Ex Vivo* Biological Evaluation of [^18^F]FPG and [^18^F]FPG-HSA

All animal procedures were carried out in accordance with the Institutional Animal Care and Use Committee Singapore (IACUC No. 181399) and conformed to the US National Institutes of Health (NIH) guidelines and public law. BALB/c mice aged 6–8 weeks were purchased from In Vivos Singapore and kept at room temperature with a 12 h light–dark cycle and had free access to food and water.

#### PET Imaging with [^18^F]FPG or [^18^F]FPG-HSA

Naïve BALB/c mice (*n* = 6) were anesthetized using a mixture of isoflurane and medical air (5% induction and 2% maintenance). The animals (*n* = 3, per group) were injected with either [^18^F]FPG (10 ± 5 MBq) or [^18^F]FPG-HSA (9 ± 1 MBq) via the lateral tail vein. Dynamic PET scans of 120 min were immediately performed by using an Inveon microPET/CT scanner (Siemens). During scanning, animals were kept on electronic heating pads and monitored for body temperature and respiration rate using a Biovet physiological monitoring system. PET images were corrected for decay and scatter and iteratively reconstructed to 16 frames (5 × 60, 1 × 300, 10 × 600 s). The radioactive uptake in different organs was estimated by drawing a region of interest (ROI) delineated by CT. The ROIs were transferred from the CT template to the PET data, and regional time–activity curves were generated. The analysis of reconstructed calibrated images was performed using Amide software (version 10.3 Sourceforge), and data are expressed as percentage of injected dose per gram (% ID/g) in the ROIs.

#### *Ex Vivo* Biodistribution of [^18^F]FPG and [^18^F]FPG-HSA

Naïve BALB/C mice (*n* = 3, per time point) were injected with either [^18^F]FPG or [^18^F]FPG-HSA (∼1.0 MBq) via the lateral tail vein and sacrificed by cervical dislocation at 10, 30, or 60 min postinjection for [^18^F]FPG or 30, 60, or 120 min postinjection for [^18^F]FPG-HSA. All major organs were excised and weighed. The radioactivity in each organ was quantified using a Wallac γ counter and expressed as % ID/g. The total injected dose was defined as the sum of the whole body counts, excluding the tail.

## Supporting Information Available

Stability tests of [^18^F]FPG in 2% DMSO/PBS at 37 °C for 4 h (Figure S1); HPLC chromatograms of [^18^F]FPG-bovine ubiquitin coinjected with bovine ubiquitin (Figure S2); HPLC chromatograms of [^18^F]FPG-methylated bovine ubiquitin coinjected with methylated bovine ubiquitin (Figure S3); HPLC chromatograms of [^18^F]FPG-IL-2 coinjected with IL-2 (Figure S4); HPLC chromatograms of [^18^F]FPG-IL-4 coinjected with IL-4 (Figure S5); (A) ESI-MS of bovine ubiquitin; (B) ESI-MS of 4-FPG-bovine ubiquitin; and (C) plausible chemical structures of the bioconjugated moiety between 4-FPG and arginine (Figure S6); time–activity curves of [^18^F]FPG in healthy mice (mean ± SD, *n* = 3) in 120 min (Figure S7); time–activity curves of [^18^F]FPG-HSA in healthy mice (mean ± SD, *n* = 3) in 120 min (Figure S8); and biodistribution of [^18^F]FPG at 10, 30, or 60 min and [^18^F]FPG-HSA at 30, 60, or 120 min post IV injection (*n* = 3, per time point) (Table S1) (PDF)

## Abbreviations Used

[^18^F]FPG: 4-[^18^F]fluorophenylglyoxal
HSA: human serum albumin
IL-2: interleukin-2
IL-4: interleukin-4
pH 7.4 PBS: 0.1 M phosphate-buffered saline
PET: positron emission tomography
p*K*_a_: acid dissociation constant
