# Supplementary material for: Arginine-Selective Bioconjugation Reagent for Effective 18F-labeling of Native Proteins
Source: J Med Chem. 2024 Mar 13;67(6):5064–74. doi: 10.1021/acs.jmedchem.4c00154 (PMC10982996; doi:10.1021/acs.jmedchem.4c00154)
Supplement: Supplementary file 1 — jm4c00154_si_001.pdf [file jm4c00154_si_001.pdf]

Supporting information

An Arginine Selective Bioconjugation Reagent for Effective ^{18}F-labeling of Native Proteins

Authors: Pragalath Sadasivam[1,2,3,6], Shivashankar Khanapur[2], Siddesh V Hartimath[2], Boominathan Ramasamy[2], Peter Cheng[2], Chin Zan Feng[2], David Green[3], Christopher Davis[1], Julian L Goggi[2,6], Edward G Robins[2,3,4,5], Ran Yan[1*]

[1]School of Biomedical Engineering and Imaging Sciences, Department of Imaging Chemistry and Biology, King's College London, UK.

[2]Institute of Bioengineering and Bioimaging, Agency for Science, Technology, and Research (A* STAR), 11 Biopolis Way, #01-02 Helios, Singapore 138667, Singapore.

[3]Clinical Imaging Research Centre, 14 Medical Drive, #B01-01 Centre for Translational Medicine, Yong Loo Lin School of Medicine, National University of Singapore, Singapore 117599, Singapore.

[4]Molecular Imaging and Therapy Research Unit, South Australian Health and Medical Research Institute (SAHMRI), North Terrace, Adelaide SA 5000, Australia.

[5]Adelaide Medical School, Faculty of Health and Medical Sciences, University of Adelaide, North Terrace & George Street, Adelaide SA 5000, Australia.

[6]Minerva Imaging ApS, Lyshøjvej 21, 3650 Ølstykke, Denmark.

Corresponding Author's email address: ran.yan@kcl.ac.uk

Table of contents

Figure S1. Stability tests of the [^{18}F]FPG in 2% DMSO/PBS at 37 °C for 4 h S2

Figure S2. HPLC chromatograms of [^{18}F]FPG-bovine ubiquitin con-injected with bovine ubiquitin. S2

Figure S3. HPLC chromatograms of [^{18}F]FPG-methylated bovine ubiquitin co-injected with methylated bovine ubiquitin. S2

Figure S4. HPLC chromatograms of [^{18}F]FPG-IL-2 co-injected with IL-2. S3

Figure S5. HPLC chromatograms of [^{18}F]FPG-IL4 co-injected with IL-4. S3

Figure S6. A) ESI-MS of bovine ubiquitin; **B**) ESI-MS of 4-FPG-bovine ubiquitin; **C**) plausible chemical structures of the bioconjugated moiety between 4-FPG and arginine. S3

Figure S7. The time-activity curves of [^{18}F]FPG in healthy mice (mean±SD, n = 3) in 120 min. S4

Figure S8. The time-activity curves of [^{18}F]FPG-HSA in healthy mice (mean±SD, n = 3) in 120 min. S4

Table S1. Biodistribution of [^{18}F]FPG at 10, 30, or 60 min and [^{18}F]FPG-HSA at 30, 60, or 120 min post IV injection (n = 3, per timepoint). S5

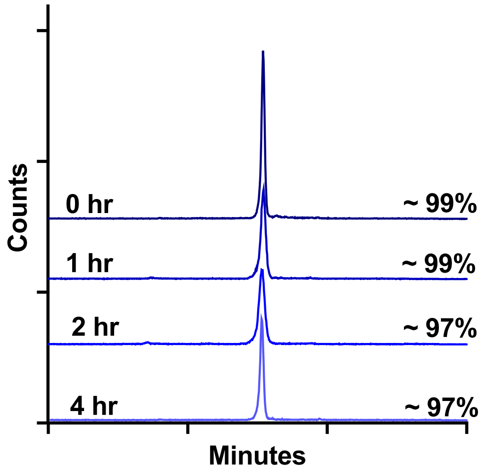

Figure S1. Stability tests of the [^{18}F]FPG in 2% DMSO/PBS at 37 °C for 4 h.

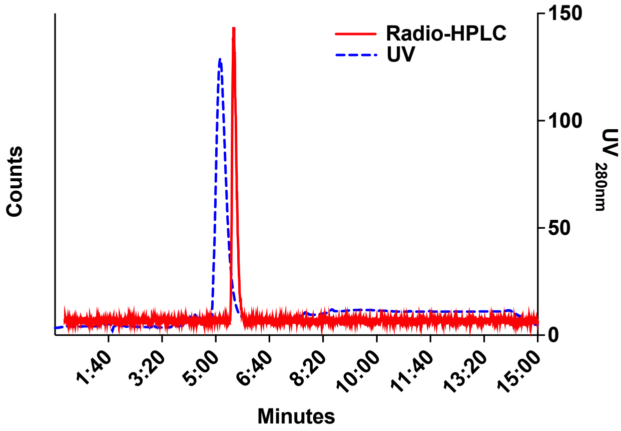

Figure S2. HPLC chromatograms of [^{18}F]FPG-bovine ubiquitin co-injected bovine ubiquitin.

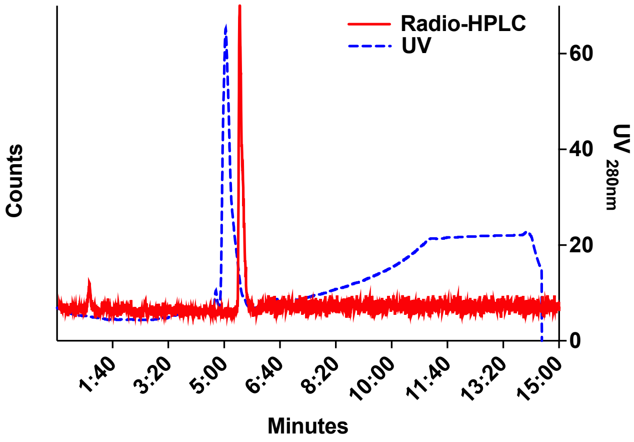

Figure S3. HPLC chromatograms of [^{18}F]FPG-methylated bovine ubiquitin co-injected with methylated bovine ubiquitin.

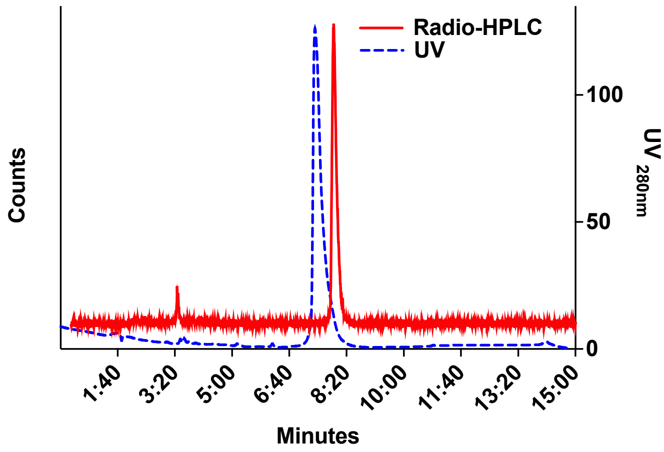

Figure S4. HPLC chromatograms of [^{18}F]FPG-IL-2 co-injected with IL-2.

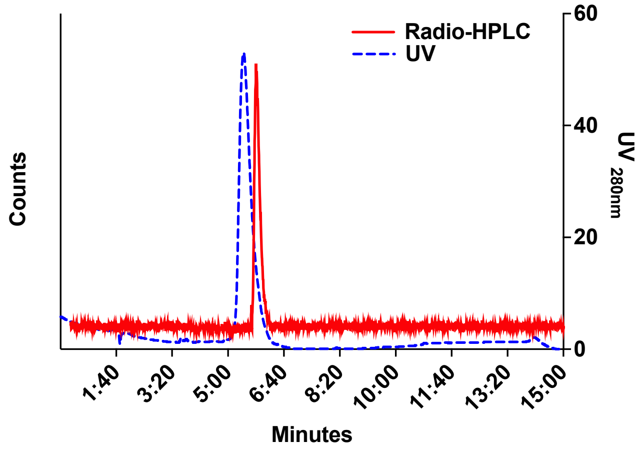

Figure S5. HPLC chromatograms of [^{18}F]FPG-IL4 co-injected with IL-4.

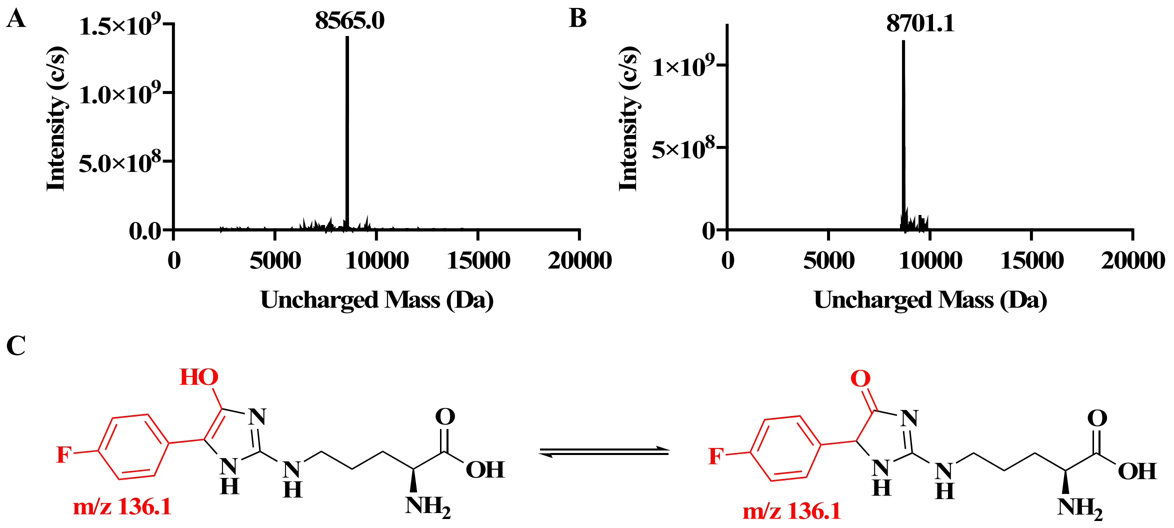

Figure S6. A) ESI-MS of bovine ubiquitin; **B**) ESI-MS of 4-FPG-bovine ubiquitin; **C**) plausible chemical structures of the bioconjugated moiety between 4-FPG and arginine.

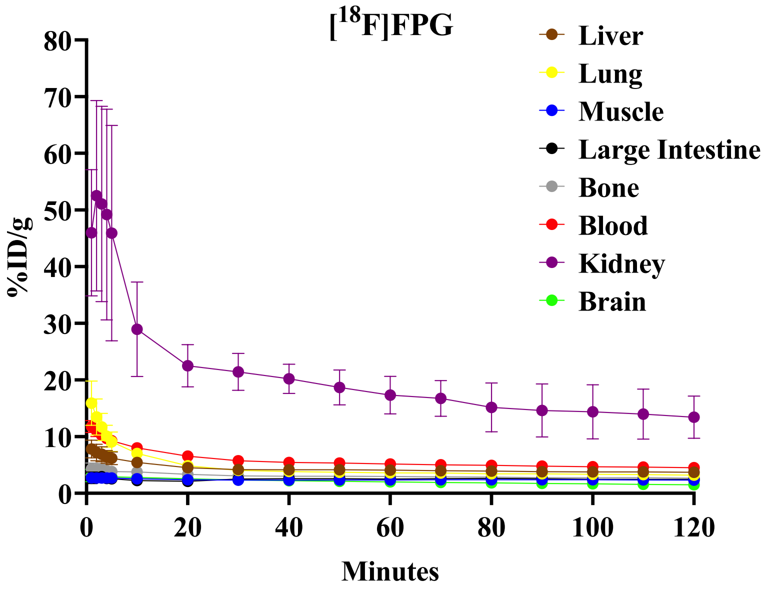

Figure S7. The time-activity curves of [^{18}F]FPG in healthy mice (mean±SD, n = 3) in 120 min.

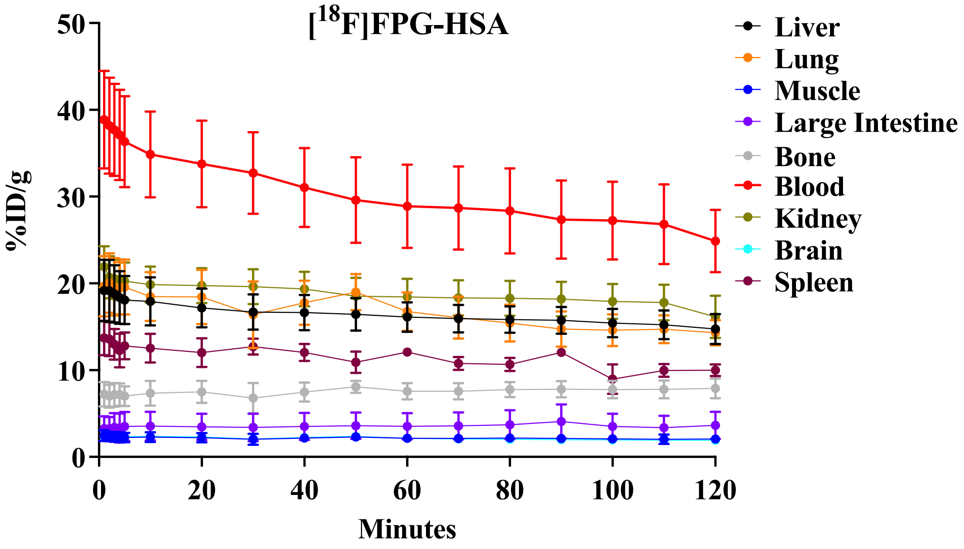

Figure S8. The time-activity curves of [^{18}F]FPG-HSA in healthy mice (mean±SD, n = 3) in 120 min.

Table S1. Biodistribution of [^{18}F]FPG at 10, 30, and 60 min and [^{18}F]FPG-HSA at 30, 60, and 120 min post IV injection (n = 3, per timepoint).

**[^{18}F]FPG *ex vivo* biodistribution (%ID/g)**			
**Organ**	**10 min**	**30 min**	**60 min**
Bone	2.6 ± 0.5	1.4 ± 0.3	0.6 ± 0.1
Muscle	2.7 ± 0.7	1.8 ± 0.4	0.4 ± 0.1
Blood	7.6 ± 0.8	2.0 ± 0.4	0.9 ± 0.2
Lung	11.8 ± 0.9	1.6 ± 0.2	0.8 ± 0.2
Liver	6.1 ± 0.4	3.0 ± 0.4	1.8 ± 0.3
Thymus	3.0 ± 0.2	1.0 ± 0.3	0.4 ± 0.1
Spleen	4.5 ± 0.5	1.3 ± 0.4	0.5 ± 0.1
Skin	4.0 ± 0.5	1.5 ± 0.3	0.4 ± 0.1
Kidney	30.4 ± 3.3	6.4 ± 1.1	3.1 ± 0.3
Heart	5.6 ± 0.6	1.6 ± 0.2	0.7 ± 0.1
Brain	2.0 ± 0.2	1.2 ± 0.1	0.5 ± 0.1

**[^{18}F]FPG-HSA *ex vivo* biodistribution (%ID/g)**			
**Organ**	**30 min**	**60 min**	**120 min**
Bone	4.0 ± 1.8	4.9 ± 0.4	4.4 ± 0.7
Muscle	2.1 ± 1.2	1.4 ± 0.4	1.5 ± 0.4
Blood	46.4 ± 3.7	39.6 ± 1.4	35.8 ± 2.9
Lung	15.5 ± 4.4	12.6 ± 0.4	9.2 ± 3.8
Liver	14.9 ± 1.2	15.1 ± 0.7	13.7 ± 2.2
Thymus	3.8 ± 1.7	3.3 ± 0.1	2.9 ± 1.4
Spleen	8.7 ± 2.8	10.7 ± 0.5	6.3 ± 0.6
Skin	2.6 ± 0.4	2.4 ± 0.6	5.1 ± 1.8
Kidney	13.1 ± 1.2	13.8 ± 2.0	14.0 ± 2.2
Heart	10.5 ± 1.8	11.0 ± 0.9	11.2 ± 1.2
Brain	1.0 ± 0.2	0.9 ± 0.2	1.0 ± 0.2
